# Exploring causality of the association between smoking and Parkinson’s disease

**DOI:** 10.1093/ije/dyy230

**Published:** 2018-11-20

**Authors:** Valentina Gallo, Paolo Vineis, Mariagrazia Cancellieri, Paolo Chiodini, Roger A Barker, Carol Brayne, Neil Pearce, Roel Vermeulen, Salvatore Panico, Bas Bueno-de-Mesquita, Nicola Vanacore, Lars Forsgren, Silvia Ramat, Eva Ardanaz, Larraitz Arriola, Jesper Peterson, Oskar Hansson, Diana Gavrila, Carlotta Sacerdote, Sabina Sieri, Tilman Kühn, Verena A Katzke, Yvonne T van der Schouw, Andreas Kyrozis, Giovanna Masala, Amalia Mattiello, Robert Perneczky, Lefkos Middleton, Rodolfo Saracci, Elio Riboli

**Affiliations:** 1 Centre for Primary Care and Public Health, Blizard Institute, Queen Mary University of London, London, UK; 2 School of Public Health, Imperial College London, London, UK; 3 Epidemiology and Medical Statistics Unit, London School of Hygiene and Tropical Medicine, London, UK; 4 School of Hygiene and Preventive Medicine, University of Campania ‘Luigi Vanvitelli’, Naples, Italy; 5 Hygiene and Public Health Unit, Department of Public Health, AUSL Imola, Bologna, Italy; 6 Medical Statistics Unit, University of Campania ‘Luigi Vanvitelli’, Naples, Italy; 7 Institute of Public Health, University of Cambridge, Cambridge, UK; 8 Julius Center for Health Sciences and Primary Care, University Medical Center Utrecht, Utrecht, The Netherlands; 9 Division of Epidemiology, Institute for Risk Assessment Science, Utrecht University, Utrecht, The Netherlands; 10 Dipartimento di Medicina Clinica e Chirurgia, Federico II University, Naples, Italy; 11 National Institute for Public Health and the Environment, Bilthoven, The Netherlands; 12 Department of Gastroenterology and Hepatology, University Medical Centre, Utrecht, The Netherlands; 13 Department of Social and Preventive Medicine, Faculty of Medicine, University of Malaya, Kuala Lumpur, Malaysia; 14 National Centre for Disease Prevention and Health Promotion, Italian National Institute of Health, Rome, Italy; 15 Department of Pharmacology and Clinical Neuroscience, Umeå University, Umeå, Sweden; 16 Department of Neuroscience, Psychology, Drug Research, and Child Health, University of Florence, Careggi Hospital-University, Florence, Italy; 17 Navarra Public Health Institute, IdiSNA, Pamplona, Spain; 18 CIBER Epidemiology and Public Health, CIBERESP, Madrid, Spain; 19 Public Health Department of Gipuzkoa, Basque Government, Vitoria-Gasteiz, Spain; 20 Biodonostia Research Institute, Neurosciences Area, Hospital Universitario Donostia, Donostia, Spain; 21 Department of Neurology, Lund University, Lund, Sweden; 22 Clinical Memory Research Unit, Department of Clinical Sciences Malmö, Lund University, Lund, Sweden; 23 Department of Epidemiology, Murcia Regional Health Council, IMIB-Arrixaca, Murcia, Spain; 24 Unit of Cancer Epidemiology, Centre for Cancer Prevention (CPO-Piemonte), Turin, Italy; 25 Human Genetic Foundation (HuGeF), Turin, Italy; 26 Epidemiology and Prevention Unit, Fondazione IRCCS Istituto Nazionale dei Tumori, Milan, Italy; 27 Division of Cancer Epidemiology, German Cancer Research Centre (DKFZ), Heidelberg, Germany; 28 Hellenic Health Foundation, Athens, Greece; 29 First Department of Neurology, University of Athens, Athens, Greece; 30 Cancer Risk Factors and Lifestyle Epidemiology Unit, Institute for Cancer Research, Prevention, and Clinical Network (ISPRO), Florence, Italy; 31 Department of Psychiatry and Psychotherapy, Ludwig-Maximilians-Universität München, Munich, Germany; 32 German Centre for Neurodegenerative Disorders (DZNE), Munich, Germany; 33 Munich Cluster for System Neurology (SyNergy), Munich, Germany; 34 International Agency for Research on Cancer (IARC), Lyon, France

**Keywords:** Parkinson’s disease, smoking, smoking patterns, passive smoking, causal inference, cohort study, EPIC, NeuroEPIC4PD

## Abstract

**Background:**

The aim of this paper is to investigate the causality of the inverse association between cigarette smoking and Parkinson’s disease (PD). The main suggested alternatives include a delaying effect of smoking, reverse causality or an unmeasured confounding related to a low-risk-taking personality trait.

**Methods:**

A total of 715 incident PD cases were ascertained in a cohort of 220 494 individuals from NeuroEPIC4PD, a prospective European population-based cohort study including 13 centres in eight countries. Smoking habits were recorded at recruitment. We analysed smoking status, duration, and intensity and exposure to passive smoking in relation to PD onset.

**Results:**

Former smokers had a 20% decreased risk and current smokers a halved risk of developing PD compared with never smokers. Strong dose–response relationships with smoking intensity and duration were found. Hazard ratios (HRs) for smoking <20 years were 0.84 [95% confidence interval (CI) 0.67–1.07], 20–29 years 0.73 (95% CI 0.56–0.96) and >30 years 0.54 (95% CI 0.43–0.36) compared with never smokers. The proportional hazard assumption was verified, showing no change of risk over time, arguing against a delaying effect. Reverse causality was disproved by the consistency of dose–response relationships among former and current smokers. The inverse association between passive smoking and PD, HR 0.70 (95% CI 0.49–0.99) ruled out the effect of unmeasured confounding.

**Conclusions:**

These results are highly suggestive of a true causal link between smoking and PD, although it is not clear which is the chemical compound in cigarette smoking responsible for the biological effect.

**Key Messages**

- The present data from the NeuroEPIC4PD study show a robust inverse association between smoking status at recruitment and Parkinson’s disease (PD) risk with a dose–response relationship with smoking duration and intensity.
- These inverse relationships were replicated across different clinical subtypes.
- An inverse association between exposure to passive smoking at home and/or at work and risk of PD was also identified.
- Explanation alternatives to a causal association including a delaying effect of smoking on disease onset, reverse causality, and unmeasured and residual confounding have been discussed in order to reinforce causal inference using observational data.

## Background

An overwhelming amount of evidence exists on the inverse association between cigarette smoking and Parkinson’s disease (PD). The inverse association is strong and consistent across studies,^1^ stronger for current smokers than for former smokers when compared with non-smokers.^1^^,^^2^ Some studies suggest that smoking duration is more strongly associated with a reduced risk of PD compared with smoking intensity.^3^ The overall association appears consistent in men and women^1^ and not confounded or modified by educational level. A comparable inverse association was also observed for pipe and cigar smoking in men^4^ and for smokeless tobacco.^5^^,^^6^ An attempt to demonstrate causality of the association has been made using parental smoking as an instrumental variable: it was shown that children of smokers—who are more likely to smoke themselves—are at decreased risk of PD even if they do not smoke.^7^

**Figure 1.**
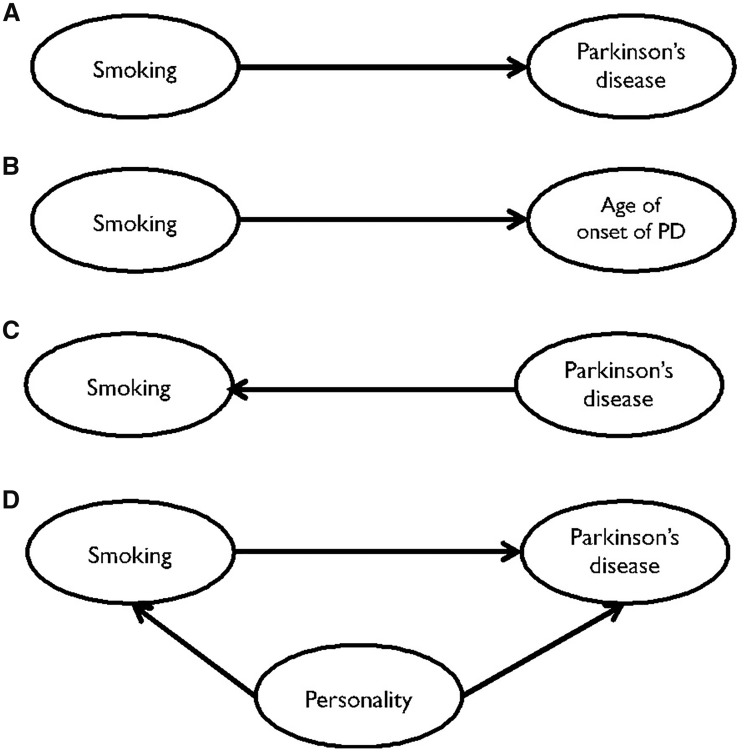
Direct Acyclic Graphs (DAGs) showing the hypotheses on the observed association between cigarette smoking and Parkinson’s disease. (**A**) Smoking protects against PD (causal effect); (**B**) smoking delays PD onset; (**C**) subjects with a specific personality trait are both less likely to smoke and more susceptible to PD (confounding effect); (**D**) subtle dopaminergic changes before disease onset make quitting smoking easier (reverse causality).

Nonetheless, there is still considerable caution in interpreting this association as protective. Few theories have been postulated to explain the current evidence in a non-causal way and these are summarized with Direct Acyclic Graphs (DAGs) in Figure 1. Some studies failed to replicate the association in cases with an older age of onset^3^^,^^8^ leading to the hypothesis that smoking might delay, not prevent, PD onset (Figure 1B). The most intriguing, and more difficult to prove, is a possible confounding effect by a low-risk-taking personality trait that would be regarded as an unmeasured confounder if it is genetically determined or as reverse causation if it is triggered by dopamine shortage^9^^,^^10^ (Figure 1C and D). According to this, and coherently with the involvement of dopamine in the brain-rewarding circuits,^11^ people who will subsequently develop PD tend to have a low-risk-taking personality, which makes them less likely to smoke or more likely to quit. Coherently, before disease onset, people with PD might find it easier to quit smoking compared with those without PD^12^ (Figure 1D). Nonetheless, the inverse association between smoking intensity and PD observed among monozygotic twins argues against a major role of genetics and/or personality.^13^ Given that personality trait would have a lesser role in influencing the exposure to passive smoking, demonstrating a decreased risk of PD among those exposed to passive smoking would overcome this effect; however, a previous study failed to find it.^14^

Clarifying the causal nature of the association between smoking and PD would contribute to understanding the mechanisms underlying the disease, informing potential targets for preventive or early treatments. Moreover, no data are currently available on the consistency of the inverse association between smoking and PD across clinical subtypes.

The aim of this study is to assess the association between smoking patterns (duration, amount and time since quitting smoking) and PD risk. Specifically, the potential delaying effect; the consistency of smoking patterns among current and former smokers to interrogate any reverse causality; the association with passive smoking; and the consistency of the association across clinical subtypes will be investigated.

## Methods

### Population

The NeuroEPIC4PD study involved 220 494 subjects recruited in Sweden, the UK, the Netherlands, Germany, Spain, Italy and Greece from the general population residing in defined geographical areas between 1992 and 2002 and aged 37–70 years, within the European Presepctive Investigation into Cancer and Nutrition (EPIC) study.^15^ Exception was the Utrecht cohort, which was based on breast-cancer-screening participants.^15^ The Naples and Utrecht cohorts were restricted to women, whereas all other cohorts involved both sexes. To date, follow-up is 98.5% complete and the median follow-up time of this sample is 12.8 years [inter-quartile range (IQR) 11.5–14.2].

### Case ascertainment and sample size

A total of 881 PD cases was ascertained in the participating EPIC centres.^16^ The present analysis has been conducted on a total sample of 214 533 subjects (including 715 incident PD cases) after removing 147 prevalent PD cases, 5359 subjects (including 19 PD cases) with missing information on smoking status at recruitment. Moreover, 221 subjects with PD-like conditions [Multi-System Atrophy (MSA) *N* = 24; Progressive Sopra-nuclear Palsy (PSP), *N* = 21; vascular parkinsonism, *N* = 34; Lewy Body Dementia (LBD), *N* = 34; essential tremor, *N* = 27; PD with essential tremor, *N* = 9; and unclassified parkinsonism, *N* = 72] were also removed from the analysis. The sample resulted in a total of 2 666 206 person/years. Procedures for PD case ascertainment in the EPIC cohort have been described elsewhere.^16^ In brief, in each centre, potential cases were identified through record linkage and validated through clinical record review by a neurologist expert in movement disorder who collected additional clinical data, including age of onset (defined as age when the first motor symptom was noticed) and clinical subtype at onset (tremor-dominant, postural instability/gait disturbance, akinetic-rigid forms).^16^

### Smoking characteristics

Answers to a number of questions on present and past smoking habits were collected at recruitment in the EPIC study. These included smoking status at recruitment (never, former and current smoker), age when they started smoking and quit, and number of cigarettes/day smoked at different ages. This latter information was not collected in Sweden, which was therefore excluded from all analyses on smoking intensity (*n* = 53 291). Starting from this core information, a number of variables were derived: duration of smoking (never smokers, smokers for <20, 20–29, 30+ years) missing for 4620 individuals; smoking intensity as mean lifetime cigarettes/day (never smokers, <12, 12+ cigarettes/day) missing for 10 876 individuals; time since quitting smoking, namely number of years elapsed from quitting smoking and recruitment to the cohort (never smoker, 19+, 9–18, <9 years) missing for 2221 individuals; age when quit smoking (never smoker, <33, 34–43, 44+ years) missing for 2221 individuals; and age when started smoking (never smoker, 20+, 17–19, <16 years) missing for 3011 individuals. Information on second-hand smoke (SHS) exposure was available only in a few centres: participants were asked whether any of their parents smoked when they were children in Italy, the Netherlands and Sweden (*N* = 59 329), whereas information on current SHS exposure at home or work was available only for participants recruited in Italy and Sweden (*N* = 40 816).

Additional information collected at baseline and relevant for this analysis is the highest educational level attained (none/primary, technical, secondary, university).

### Statistical analysis

Cox-regression models using age as the underlying time variable, adjusted for level of education and sex, and stratified for centre and age at recruitment, were run in order to investigate the effects of the main smoking variables in relation to PD onset. Models investigating smoking status, duration and amount of smoking, time and age since quitting smoking for former smokers and age when started smoking were investigated and *p*-values for trend across categories calculated where appropriate. Analyses were repeated using never smokers as the reference category where appropriate, in men and women separately, and restricted to tremor-dominant and akinetic-rigid forms of PD at onset. Heterogeneity across country was tested using the approach proposed by Smith *et al.*^17^ Heterogeneity was assessed by the likelihood ratio of two stratified models: one with country-specific estimates and one with overall estimates. Under the null hypothesis of no heterogeneity, this statistic follows approximately a chi-square distribution on (*k* – 1)*(*j* – 1) degrees of freedom (where *k* is the number of categories of smoking variable and *j* is the total number of countries).

In order to investigate a potential delaying effect of smoking on PD onset, possible non-proportionality was assessed using the Schoenfeld residuals.^18^ Also, the analysis on the main three smoking variables was repeated on the mid-age of PD onset after excluding subjects with an onset at 70+ years (<70 years, *N* = 385) or on late PD onset, after excluding those with an age of onset younger than 70 years (70+ years, *N* = 330). Studying separately subjects with a young age at onset (≤50 years) was not possible, as there were only 12 such cases.

For indirectly exploring reverse causality, the Cox regression exploring the dose–response relationships between smoking intensity and duration were repeated among current and former smokers at recruitment separately.

Both variables on SHS (in infancy and at recruitment) where studied in relation to PD onset in Cox-regression models repeated in never smokers only in an attempt to overcome unmeasured and residual confounding of the main association.

Finally, for exploring the possible competing risk of mortality in the smoker group, a competing-risk survival analysis was carried out using death as a competing event and the Fine and Gray regression model.^19^

A sensitivity analysis was conducted repeating the main Cox models using definite and very likely PD diagnosis only (389 PD cases). For further detail on how cases were labelled, please refer to the methodological paper.^16^ All analyses were done using STATA 12 IC and R version 3.3.2 (R Foundation for Statistical Computing, Vienna, Austria).

No direct patient involvement was needed to run this study, which was based on data previously collected.

## Results

Demographic characteristics and smoking habits for men and women in the EPIC cohort and PD cases are described in Table 1. Former smokers at recruitment had a ∼20% reduced risk of developing PD during follow-up compared with never smokers; current smokers had a halved risk compared with never smokers (Table 2). These results were highly consistent in men and women (Table 3) and no heterogeneity was detected across countries (Table 4). The difference in incidence rates across countries is more likely due to local differences in case-ascertainment procedures rather than true difference in incidence, as discussed in.^16^

**Table 1.** Demographic characteristics and smoking habits among men and women with and without PD at recruitment in the EPIC Study

	Total		Men		Women	
	*N* = 214 533		*N* = 80 389		*N* = 134 144	
	PD	Cohort	PD	Cohort	PD	Cohort
	*N* = 715	*N* = 213 818	*N* = 366	*N* = 80 023	*N* = 349	*N* = 133 795
**Age at recruitment, mean (SD)**	61.4 (8.3)	53.0 (10.0)	61.7 (8.3)	53.1 (10.1)	61.3 (8.3)	53.0 (9.9)
**Age at onset, mean (SD)** ^a^	67.5 (7.9)		67.6 (7.8)		67.3 (8.0)	
**Smoking status at recruitment**						
Never smoker, %	402 (56.2)	101 958 (47.7)	149 (40.7)	26 969 (33.7)	253 (72.5)	74 989 (56.1)
Former smoker, %	232 (32.5)	59 653 (27.9)	165 (45.1)	29 976 (37.5)	67 (19.2)	29 677 (22.2)
Current smoker, %	81 (11.3)	52 207 (24.4)	52 (14.2)	23 078 (28.8)	29 (8.3)	29 129 (21.8)
**Duration of smoking** ^b^						
<20 years, %	92 (32.4)	36 243 (33.8)	57 (28.6)	15 013 (29.6)	35 (41.2)	21 230 (37.6)
20–29 years, %	69 (24.3)	32 425 (30.2)	47 (23.6)	15 171 (29.9)	22 (25.9)	17 254 (30.5)
30+ years, %	123 (43.3)	38 601 (36.0)	95 (47.7)	20 551 (40.5)	28 (32.9)	18 050 (31.9)
**Lifetime cigarettes/day** ^c^						
<12 cigarettes/day, %	91 (50.3)	35 132 (47.8)	56 (41.5)	11 085 (31.2)	35 (76.1)	24 047 (63.4)
12+ cigarettes/day, %	90 (49.7)	38 370 (52.2)	79 (58.5)	24 478 (68.8)	11 (23.9)	13 892 (36.6)
**Time since quitting smoking** ^d^						
19+ years, %	110 (50.7)	19 737 (34.4)	82 (52.9)	10 151 (35.3)	28 (45.2)	9586 (33.5)
9–18 years, %	58 (26.7)	19 295 (33.6)	40 (25.8)	9773 (33.9)	18 (29.0)	9522 (33.2)
<9 years, %	49 (22.6)	18 415 (32.1)	33 (21.3)	8874 (30.8)	16 (25.8)	9541 (33.0)
**Age when quit smoking** ^d^						
<33 years, %	54 (24.9)	18 330 (31.9)	44 (28.4)	8 354 (29.0)	10 (16.1)	9 976 (34.8)
33–43 years, %	53 (24.4)	19 086 (33.2)	33 (21.3)	9809 (34.1)	20 (32.3)	9277 (32.4)
44+ years, %	110 (50.7)	20 031 (34.9)	78 (50.3)	10 635 (369)	32 (51.6)	9396 (32.8)
**Age when started smoking** ^e^						
20+ years, %	136 (46.0)	43 194 (36.7)	75 (36.1)	17 192 (33.3)	61 (69.3)	26 002 (45.4)
17–19 years, %	74 (25.0)	31 984 (29.4)	61 (29.3)	14 975 (29.0)	13 (14.8)	17 009 (29.7)
<16 years, %	86 (29.1)	33 688 (30.9)	72 (34.6)	19 458 (37.7)	14 (15.9)	14 230 (24.9)
**Educational level** ^f^						
None/primary, %	389 (56.1)	94 988 (44.8)	192 (54.1)	33 823 (42.7)	197 (58.3)	61 165 (46.1)
Technical, %	148 (21.4)	46 407 (21.9)	73 (20.6)	18 173 (22.9)	75 (22.2)	28 234 (21.3)
Secondary, %	69 (10.0)	33 145 (15.7)	38 (10.7)	11 788 (14.9)	31 (9.2)	21 357 (16.1)
University or above, %	87 (12.6)	37 275 (17.6)	52 (14.7)	15 463 (19.5)	35 (10.4)	21 812 (16.5)
**Passive smoking**						
In childhood^g^, %	100 (64.1)	42 491 (71.8)	36 (67.9)	8101 (66.4)	64 (62.1)	34 390 (73.2)
At home or at work^h^, %	86 (62.3)	27 941 (68.7)	34 (63.0)	9102 (74.6)	52 (61.9)	18 839 (66.1)

a 233 missing values (138 men and 85 women).

b Calculated on ever smokers only, 4620 missing values.

c Calculated on ever smokers only after excluding Swedish subjects (*N* = 53 291), 10 876 missing values.

d Calculated on former smokers only, 2221 missing values.

e Calculated on ever smokers only, 3011 missing values.

f Not including 2025 subjects with undetermined educational level.

g Available for 59 329 individuals only.

h Available for 40 816 individuals only.

**Table 2.** Cox-regression analyses showing hazard ratios (HRs) [and relative 95% confidence intervals (CIs)] and using as reference category never smokers or the appropriate category for each variable and HRs (and 95% CIs) for competing-risk models using mortality as competing risk

	PD cases	HR (95% CI)	HR (95% CI)	Competing-risk HR (95% CI)^a^
**Smoking status at recruitment**				
Never smokers	402	1.00		1.00
Former smokers	232	0.79 (0.66–0.94)		0.75 (0.63–0.89)
Current smokers	81	0.49 (0.38–0.63)		0.44 (0.35–0.57)
**Duration of smoking** ^b^				
Never smokers	402	1.00		1.00
<20 years	92	0.84 (0.67–1.07)	1.00	0.81 (0.64–1.02)
20–29 years	69	0.73 (0.56–0.96)	0.87 (0.63–1.19)	0.67 (0.51–0.87)
30+ years	123	0.54 (0.43–0.66)	0.61 (0.46–0.80)	0.49 (0.40–0.61)
		<0.001	<0.001	<0.001
**Smoking intensity** ^c^				
Never smokers	284	1.00		1.00
<12 cigarettes/day	91	0.80 (0.62–1.02)	1.00	0.77 (0.60–0.98)
12+ cigarettes/day	90	0.54 (0.42–0.71)	0.69 (0.50–0.94)	0.49 (0.38–0.64)
		<0.001	0.020	<0.001
**Time since quit smoking** ^d^				
Never smokers	402	1.00		1.00
19+ years	110	0.87 (0.69–1.09)	1.00	0.85 (0.68–1.06)
9–18 years	58	0.71 (0.53–0.95)	0.81 (0.58–1.12)	0.65 (0.49–0.87)
<9 years	49	0.68 (0.50–0.93)	0.80 (0.56–1.14)	0.65 (0.48–0.88)
		0.002	0.173	<0.001
**Age when quit smoking** ^d^				
Never smokers	402	1.00		1.00
<33 years	54	0.94 (0.70–1.26)	1.00	0.90 (0.67–1.20)
34–43 years	53	0.71 (0.52–0.95)	0.76 (0.52–1.12)	0.69 (0.51–0.93)
44+ years	110	0.74 (0.59–0.93)	0.78 (0.55–1.11)	0.69 (0.55–0.87)
		0.003	0.217	<0.001
**Age when started smoking** ^e^				
Never smokers	402	1.00		1.00
20+ years	136	0.74 (0.61–0.91)	1.00	0.70 (0.57–0.85)
17–19 years	74	0.59 (0.45–0.76)	0.76 (0.56–1.03)	0.56 (0.44–0.72)
<16 years	86	0.63 (0.49–0.81)	0.78 (0.58–1.05)	0.57 (0.45–0.73)
		<0.001	0.095	<0.001
**Passive smoking in childhood**	56	1.00		1.00
	100	0.99 (0.71–1.40)		0.97 (0.69–1.36)
		0.995		0.862
**Passive smoking at home/work**	52	1.00		1.00
	86	0.70 (0.49–0.99)		0.71 (0.50–1.01)
		0.047		0.059

a Restricted to the whole cohort except Sweden.

b Calculated after excluding 4620 (of which 29 PD) missing values.

c Calculated after excluding 10 876 missing values (of which 55 PD cases).

d Calculated after excluding 54 509 (of which 96 PD cases) missing values.

e Calculated after excluding 3011 (of which 17 PD cases) missing values.

**Table 3.** Hazard ratios (HRs) and relative 95% confidence intervals (CIs) from Cox-regression models investigating smoking variables in relation to PD onset in men and women separately and sensitivity analysis including only definite and very likely PD cases

	Men		Women		All	
	PD cases	HR (95% CI)^a^	PD cases	HR (95% CI)^a^	Definite and very likely PD cases	HR (95% CI)^a^
**Smoking status at recruitment**						
Never smokers	149	1.00	253	1.00	228	1.00
Former smokers	165	0.77 (0.62–0.97)	67	0.80 (0.60–1.07)	121	0.85 (0.66–1.08)
Current smokers	52	0.49 (0.35–0.67)	29	0.46 (0.31–0.69)	40	0.42 (0.29–0.59)
**Duration of smoking**						
Never smokers	149	1.00	253	1.00	228	1.00
<20 years	57	0.83 (0.61–1.14)	35	0.83 (0.58–1.21)	55	0.98 (0.72–1.34)
20–29 years	47	0.76 (0.54–1.06)	22	0.68 (0.43–1.07)	33	0.64 (0.44–0.94)
30+ years	95	0.55 (0.42–0.72)	28	0.45 (0.30–0.67)	64	0.52 (0.39–0.70)
	Trend	<0.001	Trend	<0.001	Trend	<0.001
**Smoking intensity** ^b^						
Never smokers	149	1.00	253	1.00	228	1.00
<12 cigarettes/day	56	0.79 (0.57–1.10)	35	0.83 (0.58–1.25)	51	0.85 (0.61–1.19)
12+ cigarettes/day	79	0.56 (0.42–0.76)	11	0.53 (0.28–0.99)	46	0.47 (0.33–0.68)
	Trend	<0.001	Trend	0.043	Trend	<0.001
**Time since quitting smoking**						
Never smoker	149	1.00	253	1.00	228	1.00
19+ years	82	0.89 (0.67–1.18)	28	0.79 (0.53–1.19)	58	1.05 (0.77–1.44)
9–18 years	40	0.68 (0.48–0.97)	18	0.78 (0.48–1.27)	28	0.67 (0.45–1.01)
<9 years	33	0.66 (0.45–0.97)	16	0.73 (0.44–1.23)	30	0.75 (0.50–1.11)
	Trend	0.008	Trend	0.106	Trend	0.046
**Age when quitting smoking**						
Never smoker	149	1.00	253	1.00	228	1.00
<33 years	44	1.10 (0.78–1.55)	10	0.56 (0.29–1.07)	36	1.25 (0.86–1.80)
34–43 years	33	0.60 (0.41–0.88)	20	0.96 (0.60–1.53)	28	0.74 (0.49–1.11)
44+ years	78	0.72 (0.54–0.97)	32	0.77 (0.52–1.12)	52	0.73 (0.53–1.01)
	Trend	0.006	Trend	0.164	Trend	0.032
**Age when started smoking**						
Never smoker	149	1.00	253	1.00	228	1.00
20+ years	75	0.71 (0.53–0.94)	61	0.77 (0.57–1.04)	67	0.70 (0.52–0.93)
17–19 years	61	0.70 (0.51–0.95)	13	0.36 (0.20–0.64)	38	0.58 (0.41–0.84)
<16 years	72	0.63 (0.47–0.84)	14	0.58 (0.33–1.02)	52	0.73 (0.53–1.01)
	Trend	0.001	Trend	<0.001	Trend	0.006
**Passive smoking in childhood**	53	1.25 (0.70–2.24)	103	0.88 (0.60–1.32)		
**Passive smoking at home/work**	54	0.71 (0.40–1.23)	84	0.68 (0.43–1.08)		

a Models adjusted for educational level and sex (where appropriated) and stratified by centre and age at recruitment.

b Excluding Sweden (*N* = 53 291) and missing for 10 876 subjects who were excluded from this model.

**Table 4.** Hazard ratios (HRs) and relative 95% confidence intervals (CIs) from Cox-regression models investigating smoking variables in relation to PD onset in each country separately and *p*-value for heterogeneity

	Italy	Spain	UK	The Netherlands	Greece	Germany	Sweden	
**PD/total**	64/40 148	101/24 924	200/27 980	13/16 909	92/25 845	50/25 436	195/53 291	
**Incidence rate per 10 000 person/years**	1.32	3.08	5.47	0.73	3.70	1.74	2.66	

	HR (95% CI)	HR (95% CI)	HR (95% CI)	HR (95% CI)	HR (95% CI)	HR (95% CI)	HR (95% CI)	p-value

**Smoking status at recruitment**								
Never smokers	1.00	1.00	1.00	1.00	1.00	1.00	1.00	0.099
Former smokers	1.11 (0.61–2.02)	0.63 (0.33–1.22)	0.91 (0.66–1.23)	0.40 (0.11–1.48)	0.71 (0.378–1.32)	0.62 (0.34–1.16)	0.74 (0.54–1.03)	
Current smokers	0.75 (0.38–1.48)	0.66 (0.36–1.21)	0.75 (0.46–1.21)	0.27 (0.03–2.17)	0.34 (0.14–0.84)	0.24 (0.07–0.81)	0.28 (0.17–0.48)	
**Duration of smoking** ^a^								
Never	1.00	1.00	1.00	1.00	1.00	1.00	1.00	0.143
<20 years	1.58 (0.81–3.11)	0.94 (0.43–2.07)	0.74 (0.46–1.20)	0.33 (0.04–2.62)	0.50 (0.15–1.67)	0.61 (0.28–1.30)	0.89 (0.60–1.31)	
20–29 years	0.78 (0.35–1.77)	0.67 (0.29–1.51)	0.96 (0.59–1.57)	0.38 (0.05–3.06)	0.79 (0.30–2.06)	0.76 (0.32–1.77)	0.59 (0.35–0.97)	
30+ years	0.73 (0.37–1.45)	0.56 (0.30–1.05)	0.77 (0.53–1.12)	0.38 (0.08–1.80)	0.54–0.28–1.02)	0.27 (0.09–0.78)	0.31 (0.19–0.50)	
Trend	0.276	0.060	0.229	0.158	0.070	0.015	P<0.001	
**Smoking intensity** ^b^								
Never	1.00	1.00	1.00	1.00	1.00	1.00	–	0.397
<12 cigarettes/day	1.08 (0.57–2.06)	0.97 (0.53–1.77)	0.91 (0.63–1.34)	0.40 (0.11–1.52)	0.60 (0.25–1.46)	0.37 (0.15–0.91)	–	
12+ cigarettes/day	0.62 (0.28–1.37)	0.39 (0.19–0.80)	0.68 (0.45–1.00)	–	0.54 (0.29–1.01)	0.59 (0.28–1.25)	–	
Trend	0.297	0.014	0.062	0.051	0.051	0.075	–	

a Calculated after excluding 4620 (of which 29 PD) missing values.

b Calculated after excluding Sweden (*N* = 53 291) and 10 876 missing values (of which 55 PD cases).

**Figure 2.**
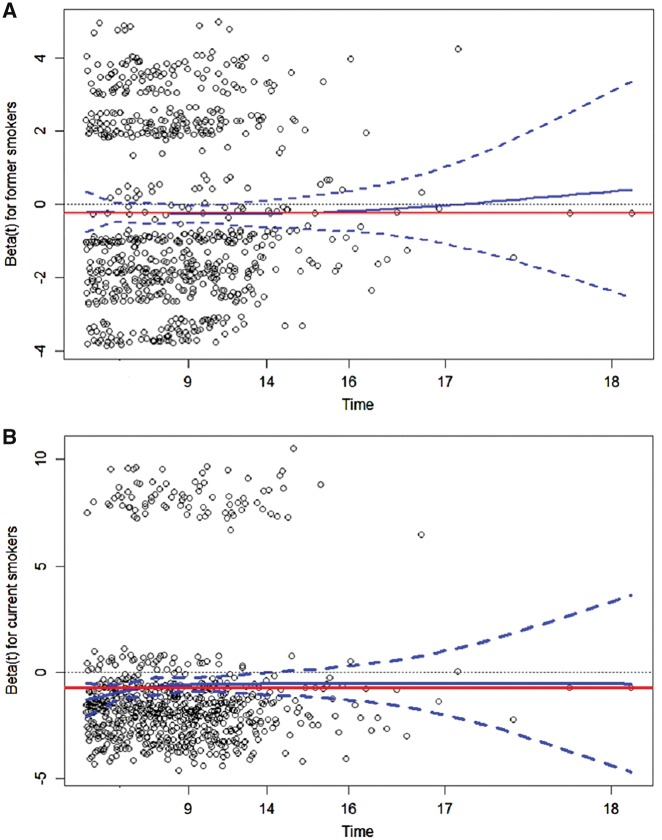
Analysis of the residuals of Schoenfeld residuals to assess the proportionality assumption comparing former smokers (**A**) and current smokers (**B**) with never smokers. Figures represent plots of beta-coefficient estimates (log hazard ratios) for former smokers (**A**) and current smokers (**B**) against follow-up (time) in years. The darker (blue) line represents a smoothed curve of scaled Shoenfeld residuals with 95% confidence intervals (darker (blue) dotted lines), whereas the lighter (red) line represents a beta-coefficient estimate from a Cox-regression model.

Studied individually, all smoking variables were found to be inversely associated with the risk of PD with clear-cut dose–response relationships. For age when started and quit smoking, a monotonic trend across categories was not evident (Table 2). The analysis of residuals of Schonefeld showed no evidence of non-proportionality over the follow-up period. The smoothed curves for former smokers (Figure 2A) and for current smokers (Figure 2B) were flat, showing that beta-coefficient (log hazard ratio) estimates did not vary during follow-up (time) (Figure 2). Smoking variables were associated with inverse risk of both mid-age and late-onset PD; however, all the estimates are stronger in the latter. All the risk estimates, conversely, remain highly consistent for the akinetic-rigid and tremor-dominant forms at onset (Table 5). The Postural Instability/Gait Disturbance (PIGD) form could not be studied individually, as it included only 42 subjects.^16^

**Table 5.** Hazard ratios (HRs) and relative 95% confidence intervals (CIs) for Cox regressions analysing risk of PD at early and older age of onset and in tremor-dominant or akinetic-rigid forms

	Mid-age PD onset		Late PD onset		Tremor-dominant PD^a^		Akinetic-rigid PD^a^	
	PD	HR	PD	HR	PD	HR	PD	HR
	(*N* = 385)	(95% CI)	(*N* = 330)	(95% CI)	(*N* = 234)	(95% CI)	(*N* = 157)	(95% CI)
**Smoking status at recruitment**								
Never smoker	215	1.00	187	1.00	140	1.00	102	1.00
Former smoker	119	0.89 (0.70–1.14)	113	0.69 (0.53–0.89)	66	0.84 (0.61–0.16)	38	0.66 (0.44–0.98)
Current smoker	51	0.51 (0.37–0.69)	30	0.48 (0.32–0.72)	28	0.47 (0.31–0.73)	17	0.39 (0.23–0.67)
**Duration of smoking**								
Never smokers	215	1.00	187	1.00	140	1.00	102	1.00
<20 years	56	0.90 (0.67–1.23)	36	0.76 (0.53–1.11)	34	1.00 (0.67–1.49)	16	0.64 (0.37–1.10)
20–29 years	37	0.68 (0.47–0.97)	32	0.81 (0.55–1.21)	25	0.82 (0.52–1.30)	11	0.49 (0.26–0.93)
30+ years	66	0.60 (0.45–0.81)	57	0.47 (0.34–0.64)	31	0.46 (0.30–0.69)	27	0.53 (0.34–0.84)
		<0.001		<0.001		<0.001		0.002
**Smoking intensity** ^b^								
Never smokers	154	1.00	130	1.00	91	1.00	62	1.00
<12 cigarettes/day	50	0.84 (0.60–1.18)	41	0.74 (0.51–1.08)	28	0.93 (0.58–1.47)	14	0.58 (0.31–1.07)
12+ cigarettes/day	55	0.62 (0.44–0.87)	35	0.46 (0.31–0.69)	20	0.46 (0.27–0.78)	18	0.50 (0.27–0.91)
		0.006		<0.001		0.007		0.014

a Information on subtype is not available for 324 PD cases.

b Restricted to the whole cohort except Sweden.

The competing-risk analysis using mortality as a competing factor yielded much stronger point estimates but largely overlapping 95% confidence intervals (CIs) for all the active smoking variables: smoking for 30+ years or 12+ cigarettes/day is associated with a ∼55% reduced risk of PD compared with never smokers (Table 2).

**Figure 3.**
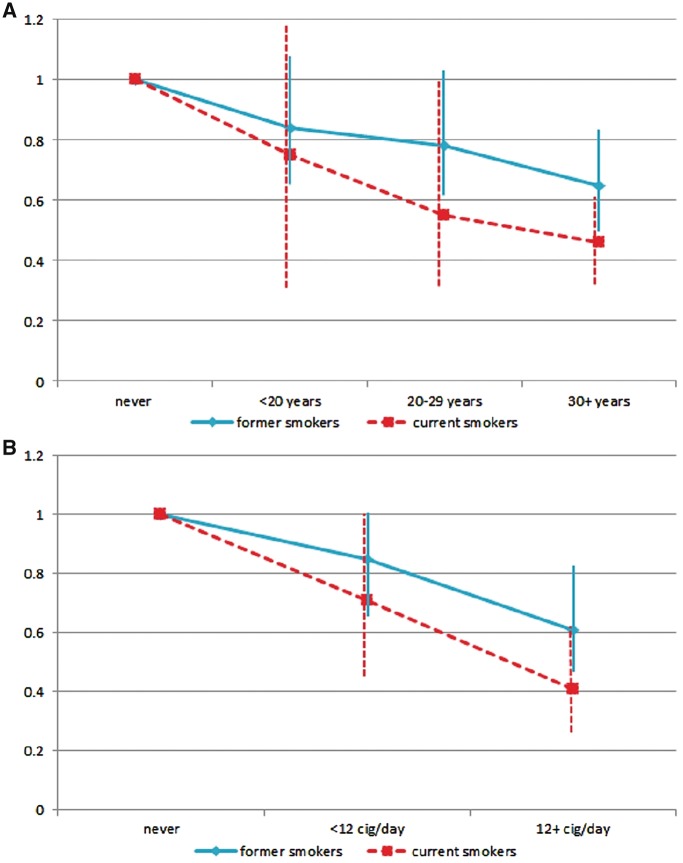
HRs and relative 95% CIs for smoking duration (**A**) and intensity (**B**) among former (continuous line) and current (dashed line) smokers at recruitment in the EPIC study.

Hazard ratios (HRs) of smoking intensity and duration from Cox models stratified for smoking status at recruitment are shown in Figure 3. Point estimates in current smokers are consistently lower compared with those in former smokers, although the pattern of risk reduction is highly comparable across the two groups, all trends had *p* ≤ 0.001 and no interaction was detected between smoking duration and intensity and smoking status (*p*-value for interaction 0.823 and 0.537, respectively).

Analysis of passive smoking, although hampered by limited power, showed no association between exposure to passive smoking in infancy and risk of PD. However, an inverse association was found between passive-smoking exposure at home or at work and risk of PD (HR 0.70, 95% CI 0.49–0.99), which was replicated among never smokers only (HR 0.71, 95% CI 0.46–1.10).

The sensitivity analysis including definite and very likely PD only yielded strikingly similar results (Table 3). All associations were, if anything, strengthened despite the widening of CIs due to the smaller sample size. An inverse association between age when quitting smoking and risk of PD was also suggested by the sensitivity analysis.

## Discussion

This study provides unique data on the inverse association between cigarette smoking and risk of PD in a large, well-established cohort study, supporting previous findings,^3^^,^^4^^,^^8^ and allows testing of explanations other than a direct protective effect. Overall, data coming from the NeuroEPIC4PD study show a robust inverse association between smoking status at recruitment and PD risk, with a dose–response relationship between PD risk and smoking duration and intensity. Of particular interest is the replication of the main findings of the inverse relationship between smoking and PD among different subtypes of the disease. This is a novel finding, as, to our knowledge, clinical subtypes have not been investigated to date in such an epidemiological setting.

### Delaying effect of smoking

The fact that proportional assumption hypothesis is verified demonstrates that the risk does not vary over the follow-up period, and this argues against a delaying effect of smoking on PD onset (Figure 1B). Moreover, at odds with some previous reports,^3^^,^^8^ our findings of an inverse relationship between smoking variables and risk of PD are not weakened when the analysis is restricted to old-age onset PD (70+ years). Taken together, these results are not supportive of the hypothesis that smoking might delay, rather than prevent, PD onset, as previously suggested.^3^^,^^8^ However, despite this piece of evidence being important and informative per se, the distinction between delaying and preventing any disease onset is somewhat artificial, as these mechanisms might coincide from both a clinical and a biological point of view.

### Reverse causality

If an inverse causal relationship—accounting for subjects with a preclinical dopaminergic change who therefore might find it easier to quit smoking—was responsible for the observed inverse association between smoking and PD, the dose–response relationship between smoking duration and intensity should not hold true among former smokers (Figure 1C). The fact that the risk of PD was reduced among current and former smokers argues against this possible explanation. Furthermore, the inverse association between time since cessation and PD reinforces the idea that reverse causality is not a likely explanation of the findings: having quit smoking 9–18 years before recruitment into the study (therefore up to 30 years before disease onset) still confers a reduced risk of PD compared with never smokers. This results are in line with previous observational studies that showed an inverse association between parental smoking and PD in the offspring;^7^ also, the use of parental smoking as an instrumental variable overcomes the potential for a reverse-causality effect.

### Unmeasured confounding

Whereas it was not possible to account for personality trait, its unmeasured confounding effect can be overcome by using exposure to passive smoking in relation to PD onset. Risk propensity is likely to influence one’s attitude towards active smoking, whereas passive smoking is more likely to be related to these personal characteristics in a weaker way (e.g. smokers tend to have smoking partners). The inverse association between passive smoking and PD onset, whose point estimate has been replicated among never smokers only, argues against considering personality trait as a major confounder. These results are in line with previous reports showing how adjusting for sensation-seeking score only slightly attenuated the inverse association between smoking and PD suggesting an independent effect^20^ and with observations that personality traits such as neuroticism and introversion do not explain the inverse association between smoking and PD risk.^21^

### Biological plausibility

A number of substances present in tobacco have been proposed as potentially responsible for the inverse association between smoking and PD. One of these is 2,3,6-trimethyl-1,4-naphthoquinone (TMN), an inhibitor of monoamine oxidase (MAO) A and B activity.^22^ TMN partially protects against 1-methyl-4-phenyl-1,2,3,6-tetrahydropyridine (MPTP)-induced neurodegeneration in mice by reducing endogenous dopamine metabolism and consequently decreasing oxidative stress. Synthetic MAO B inhibitors are currently used in the treatment of PD, providing symptomatic relief, but they may also protect against nigrostriatal damage decreasing dopamine metabolism, as suggested by delayed need for antiparkinsonian drugs in a recent clinical trial.^23^ Another candidate is nicotine itself, given the close anatomical relationship between the nicotinic cholinergic and dopaminergic neurotransmitter systems in the striatum. Nicotine influences also the dopaminergic activity by acting at nicotinic receptors on dopaminergic terminals and modulating dopamine release.^24^^,^^25^ The role of nicotine is being investigated in a randomized trial in patients with early PD, but a role of other tobacco components cannot be excluded.

Being exposed to passive smoke is associated with a reduced risk of 30% (HR 0.70, 95% CI 0.49–0.99) and being a light smoker with a 20% reduced risk (HR 0.80, 95% CI 0.62–1.02) (Table 2). Although the difference could be due to limits in the design (data on passive smoking were available for a subset of the sample), it cannot be excluded that passive smoking has a stronger effect than one would expect from a pure equivalence of levels of exposure. Passive smoking has been demonstrated to be as mutagenic as active smoking,^26^ although earlier studies suggest that the overall chemical composition of passive smoking might not represent only the diluted composition of side-stream smoking, given the sorbing and desorbing properties of some volatile and semi-volatile organic compounds in passive smoking.^27^

The main strengths of this study are the prospective design, the validated clinical outcome,^28^ the large sample and the detailed information on smoking patterns. This allowed a powered recall-bias-free analysis of smoking patterns in relation to PD onset. The main limitation of this study, however, is the lack of repeated smoking measurements over time, which might introduce some exposure misclassification, decreasing our ability to study smoking patterns in relation to PD onset. This is particularly true for outcomes ascertained many years after recruitment. However, the smoking pattern analyses repeated separately for PD cases ascertained within and after 8 years since recruitment yield highly consistent results (data not shown).

## Conclusions

In conclusion, the present findings are consistent with a protective effect of smoking on the risk of PD. Point estimates of smoking status are strong, with a strong exposure–response relationship of smoking intensity and duration. The consistency across different disease subtypes suggests that the putative protective effect might spread to the entire clinical spectrum of the disease. Finally, the inverse association found between passive smoking and PD is supported by a consistent finding among never smokers and points towards a true biological effect not mediated by personality type. Although smoking to prevent PD cannot be recommended given the multiple adverse effects of smoking, our results confirming an inverse association warrants further research on the mechanisms involved. In particular, the use of Mendelian randomization and biomarkers of long-term cigarette-smoke exposure should provide compelling final evidence on the inverse association between smoking and PD.

## Funding

No specific funding was available for this study. The researchers are independent from any funding sources with regard to this study.

## References

[1] Li X, Li W, Liu G, Shen X, Tang Y. Association between cigarette smoking and Parkinson’s disease: a meta-analysis. Arch Gerontol Geriatr 2015;61:510–16. 26272284 10.1016/j.archger.2015.08.004

[2] Checkoway H, Powers K, Smith-Weller T, Franklin GM, Longstreth WT Jr, Swanson PD. Parkinson’s disease risks associated with cigarette smoking, alcohol consumption, and caffeine intake. Am J Epidemiol 2002;155:732–38. 11943691 10.1093/aje/155.8.732

[3] Chen H, Huang X, Guo X et al Smoking duration, intensity, and risk of Parkinson disease. Neurology 2010;74:878–84. 20220126 10.1212/WNL.0b013e3181d55f38 PMC2836869

[4] Ritz B, Ascherio A, Checkoway H et al Pooled analysis of tobacco use and risk of Parkinson disease. Arch Neurol 2007;64:990–97. 17620489 10.1001/archneur.64.7.990

[5] O’Reilly EJ, McCullough ML, Chao A et al Smokeless tobacco use and the risk of Parkinson’s disease mortality. Mov Disord 2005;20:1383–84. 16007624 10.1002/mds.20587

[6] Yang F, Pedersen NL, Ye W et al Moist smokeless tobacco (Snus) use and risk of Parkinson’s disease. Int J Epidemiol 2017;46:872–80. 27940486 10.1093/ije/dyw294

[7] O’Reilly EJ, Chen H, Gardener H, Gao X, Schwarzschild MA, Ascherio A. Smoking and Parkinson’s disease: using parental smoking as a proxy to explore causality. Am J Epidemiol 2009;169:678–82. 19131566 10.1093/aje/kwn388 PMC2727210

[8] Thacker EL, O’Reilly EJ, Weisskopf MG et al Temporal relationship between cigarette smoking and risk of Parkinson disease. Neurology 2007;68:764–68. 17339584 10.1212/01.wnl.0000256374.50227.4b PMC2225169

[9] de Lau LM, Breteler MM. Epidemiology of Parkinson’s disease. Lancet Neurol 2006;5:525–35. 16713924 10.1016/S1474-4422(06)70471-9

[10] van der Mark M, Nijssen PC, Vlaanderen J et al A case-control study of the protective effect of alcohol, coffee, and cigarette consumption on Parkinson disease risk: time-since-cessation modifies the effect of tobacco smoking. PLoS One 2014;9:e95297. 24788751 10.1371/journal.pone.0095297 PMC4005732

[11] Jentsch JD, Pennington ZT. Reward, interrupted: inhibitory control and its relevance to addictions. Neuropharmacology 2014;76(Pt B):479–86. 23748054 10.1016/j.neuropharm.2013.05.022 PMC4023480

[12] Ritz B, Lee PC, Lassen CF, Arah OA. Parkinson disease and smoking revisited: ease of quitting is an early sign of the disease. Neurology 2014;83:1396–402. 25217056 10.1212/WNL.0000000000000879 PMC4206154

[13] Tanner CM, Goldman SM, Aston DA et al Smoking and Parkinson’s disease in twins. Neurology 2002;58:581–88. 11865136 10.1212/wnl.58.4.581

[14] Tanaka K, Miyake Y, Fukushima W et al Active and passive smoking and risk of Parkinson’s disease. Acta Neurol Scand 2010;122:377–82. 20175761 10.1111/j.1600-0404.2010.01327.x

[15] Riboli E, Hunt KJ, Slimani N et al European Prospective Investigation into Cancer and Nutrition (EPIC): study populations and data collection. Public Health Nutr 2002;5:1113–24. 12639222 10.1079/PHN2002394

[16] Gallo V, Brayne C, Forsgren L et al Parkinson’s disease case ascertainment in the EPIC cohort: the NeuroEPIC4PD study. Neurodegener Dis 2015;15:331–38. 26375921 10.1159/000381857

[17] Smith CT, Williamson PR, Marson AG. Investigating heterogeneity in an individual patient data meta-analysis of time to event outcomes. Stat Med 2005;24:1307–19. 15685717 10.1002/sim.2050

[18] Grambsch PM. Goodness-of-fit and diagnostics for proportional hazards regression models. Cancer Treat Res 1995;75:95–112. 7640169 10.1007/978-1-4615-2009-2_5

[19] Fine JP, Gray RJ. A proportional hazard model for the subdistribution of a competing risk. J Am Stat Assoc 1999;94:496–509.

[20] Evans AH, Lawrence AD, Potts J et al Relationship between impulsive sensation seeking traits, smoking, alcohol and caffeine intake, and Parkinson’s disease. J Neurol Neurosurg Psychiatry 2006;77:317–21. 16484638 10.1136/jnnp.2005.065417 PMC2077692

[21] Sieurin J, Gustavsson P, Weibull CE et al Personality traits and the risk for Parkinson disease: a prospective study. Eur J Epidemiol 2016;31:169–75. 26130127 10.1007/s10654-015-0062-1 PMC4819915

[22] Quik M, Perez XA, Bordia T. Nicotine as a potential neuroprotective agent for Parkinson’s disease. Mov Disord 2012;27:947–57. 22693036 10.1002/mds.25028 PMC3685410

[23] Rascol O, Fitzer-Attas CJ, Hauser R et al A double-blind, delayed-start trial of rasagiline in Parkinson’s disease (the ADAGIO study): prespecified and post-hoc analyses of the need for additional therapies, changes in UPDRS scores, and non-motor outcomes. Lancet Neurol 2011;10:415–23. 21482191 10.1016/S1474-4422(11)70073-4

[24] Grady SR, Salminen O, Laverty DC et al The subtypes of nicotinic acetylcholine receptors on dopaminergic terminals of mouse striatum. Biochem Pharmacol 2007;74:1235–46. 17825262 10.1016/j.bcp.2007.07.032 PMC2735219

[25] Quik M, Wonnacott S. alpha6beta2* and alpha4beta2* nicotinic acetylcholine receptors as drug targets for Parkinson’s disease. Pharmacol Rev 2011;63:938–66. 21969327 10.1124/pr.110.003269 PMC3186078

[26] Husgafvel-Pursiainen K. Genotoxicity of environmental tobacco smoke: a review. Mutat Res 2004;567:427–45. 15572289 10.1016/j.mrrev.2004.06.004

[27] Daisey JM. Tracers for assessing exposure to environmental tobacco smoke: what are they tracing? Environ Health Perspect 1999;107:319–27. 10350517 10.1289/ehp.99107s2319 PMC1566270

[28] Gallo V, Brayne C, Forsgren L et al Parkinson’s disease case ascertainment in the EPIC cohort: the NeuroEPIC4PD study. Neurodegener Dis 2015;15:331–38. 26375921 10.1159/000381857

